# Biological activity of alginate and its effect on pancreatic lipase inhibition as a potential treatment for obesity

**DOI:** 10.1016/j.foodhyd.2015.02.019

**Published:** 2015-07

**Authors:** David Houghton, Matthew D. Wilcox, Peter I. Chater, Iain A. Brownlee, Chris J. Seal, Jeffrey P. Pearson

**Affiliations:** a Institute for Cell and Molecular Bioscience, Medical School, Newcastle University, Catherine Cookson Building, Framlington Place, Newcastle upon Tyne NE2 4HH, United Kingdom; b Human Nutrition Research Centre, School of Agriculture, Food & Rural Development, Newcastle University, Catherine Cookson Building, Framlington Place, Newcastle upon Tyne NE2 4HH, United Kingdom

**Keywords:** Biological, Alginate, Lipase, Pancreatic, Inhibition, WHO, World Health Organisation, GI, gastrointestinal tract, MO, methyl orange, DB, dextran blue, AB, alginate bread, CB, control bread

## Abstract

Alginates are classed as a dietary fibre and have been shown to inhibit digestive enzymes *in vitro*, and therefore could be used as an obesity treatment. The current study aims to assess whether alginate in a bread vehicle maintains its inhibition properties despite cooking and digestion, and may therefore be used as a potential treatment for obesity. After 180 min in a model gut that replicates digestion in the mouth, stomach and small intestines alginate bread (AB), control bread (CB), CB with Manucol^®^ DM alginate, free DM alginate and model gut solution were collected. DM, LFR 5/60 and SF200 were heated at 37 °C and 200 °C, with DM also heated at 50, 100 and 150 °C. Samples from the model gut and heated alginate were assessed for molecular size and inhibition properties using viscosity, gel filtration and a lipase turbidity assay. AB does not significantly increase viscosity in the model gut. Viscosity of alginate reduces beyond 100 °C, although alginate retains its inhibition properties up to 150 °C. Cooking into the bread does not reduce the molecular size of the alginate or affect its inhibition properties. These data demonstrate the robustness of alginates lipase inhibition despite the cooking process and digestion. Therefore adding alginate to a bread vehicle may have the potential in the treatment for obesity.

## Highlights

• Alginate in bread is undigested and recoverable in an *in-vitro* model gut.
• Alginate molecular size appears unaffected by cooking, digestion and extraction.
• Alginate retains its inhibition properties despite being heated at 37, 50, 100 and 150 °C.
• Alginate extracted from the model gut retains its inhibition properties.

## Graphical abstract

**Figure:**
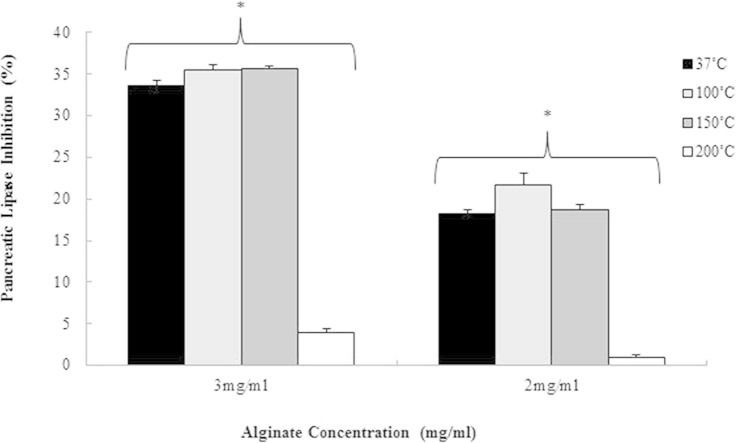

## 1 Introduction

The World Health Organisation (WHO) recognised obesity as a global epidemic in 1997, and predict that the number of people who are obese and overweight is set to continue to rise (FAO/WHO, 2003). Although the most recognised form of maintaining a healthy weight is to consume a healthy diet and exercise, this is rarely achieved with adherence rates as low as 15% (Ayyad & Andersen, 2000). Alternative weight loss treatments such as pharmaceutical agents and surgery are available, however these treatments are associated with side effects and are not cost effective (Finer, James, Kopelman, Lean, & Williams, 2000; Hadvary, Lengsfeld, & Wolfer, 1988; Pi-Sunyer, 2006; Rich, Rubin, Walker, Schneeweiss, & Abenhaim, 2000).

An alternative treatment which has received considerable interest is dietary fibre, specifically alginate as a potential weight loss treatment (Strugala, Kennington, Campbell, Skjak-Braek, & Dettmar, 2005; Sunderland, Dettmar, & Pearson, 2000; Wilcox, Brownlee, Richardson, Dettmar, & Pearson, 2014). Alginates are present as a matrix polysaccharide in the cell walls of brown algae and consist of (Grasdalen, Larsen, & Smidsrod, 1977). These residues can combine to form G rich (G blocks), M rich (M blocks) or a mixture of G and M. The pattern of residues determines the physicochemical properties of alginate. They are widely used in industry, including adding to foods or beverages as thickening and stabilising agents as reviewed by Brownlee et al. (2005). An additional benefit of alginate is that it is able to form both ionic and acidic gels. This may be a possible mechanism for a reduction in the digestibility of macronutrients and a reduction in hunger seen after consumption of alginates in mixed diets partially because of the viscosity increase caused by gel formation in the stomach at low pH (Draget, Skjåk Bræka, & Stokke, 2005; Ellis, Apling, Leeds, & Bolster, 1981; Seal & Mathers, 2001; Wolf et al., 2002).

Previous research using alginate as a potential weight loss treatment added it to a beverage or cereal bar. The benefits reported in these studies include increased satiety, reduced calories consumed, reduced blood glucose and insulin, reduced fat digestion and weight loss. These beneficial physiological effects are countered by a number of problems including poor palatability, products being returned, burping, nausea, flatulence, stomach ache and subjects preferring the control products (Georg Jensen, Kristensen, & Astrup, 2012; Sandberg et al., 1994; Torsdottir, Alpsten, Holm, Sandberg, & Tolli, 1991; Williams et al., 2004). Poor palatability of these products may be due to gel formation and a slimy mouth feel (Ellis et al., 1981). The current study developed alginate-enriched bread in an attempt to overcome these adverse side effects, although the cooking process where temperatures may exceed 180 °C (Hasatani et al., 1991) may alter alginate properties, and in doing so reduce the ability of the alginate to alter the digestion process and inhibit digestive enzymes in the upper gastrointestinal tract (GI).

There are limited data on the properties of the alginate once it has been exposed to heat above 37 °C. McDowell (1977) and Leo, McLoughlin, and Malone (1990) suggested that when polymers are heated at temperatures above 100 °C the alginate structure may depolymerise. McDowell (1977) also indicated that at extreme temperatures in excess of 200 °C complete breakdown of the alginate and a rapid evolution of CO_2_ from the uronic acid groups occurs. If these changes occurred during the cooking process the alginates may not retain their inhibitory properties in the upper GI tract. The aim of the current study was to assess whether alginate incorporated into bread retained its viscosity and inhibitory properties after baking. The study used a model gut to digest alginate-enriched bread and assess the physiochemical properties of the digesta and to determine if isolated alginate retained its inhibitory properties.

## 2 Methods

### 2.1 Materials

Sepharose 2B, methyl orange (MO), dextran blue (DB), sodium chloride, sodium azide, Tris, methanol, acetone, colipase from porcine pancreas ≥ 95% protein (Prod No: C3028), lipase from porcine pancreas type II 100–500 units/mg protein using olive oil as a substrate (Product No: L3126) and orlistat (tetrahydrolipstatin) were purchased from Sigma–Aldrich (Poole, UK). Aluminium oxide was purchased from Fisher Scientific (Loughborough, UK), and olive oil was purchased from Co-operative Foods (Manchester, UK). Deoxycholic acid sodium salt and taurodeoxycholic acid sodium salt were purchased from Fluka (Buchs, Switzerland). Alginates LFR 5/60 which is a low viscosity and low molecular weight (40,000) sodium alginate rich in guluronate F_g_ 0.64, SF200 which is a high viscosity and high molecular weight (380,000) sodium alginate rich in guluronate F_g_ 0.69 and Manucol^®^ DM which is a high viscosity sodium alginate with a molecular weight ranging from 250,000 to 320,000 were a gift from FMC BioPolymer AS, Drammen, Norway and were stored at 4 °C in tightly-sealed containers and all alginate weights were corrected for water content. The control bread (CB) and alginate bread (AB) were produced by Greggs Plc and ingredients are presented in Table 1. The AB was 4% alginate MANUCOL^®^ DM (w/w) wet dough as this made the most palatable bread.

### 2.2 Model gut procedure

The model gut replicates digestion in the mouth, stomach and the duodenum, which is consistent with criteria set out by Wickham, Faulks, and Mills (2009). In summary 5.2 g of the alginate bread (AB) and control bread (CB) were broken into crumbs ranging between 2 and 4 cm as this amount of bread and conditions that simulates the amount of bread consumed in an individual bite and mastication in the mouth. Samples were then placed into water bath two and mixed at 75 revolutions (rpm)/min for 30 s 50 ml of synthetic gastric juice was added to each sample and mixing continued for 60 min. Following this 25 ml of porcine bile was added and synthetic pancreatic juice was pumped in whilst mixing continued for an additional 120 min. Water baths, enzymes, synthetic solutions and bile were all added fresh and were set at 37 °C throughout the whole process. To ensure the model gut was replicating the pH of in-vivo digestion the pH was monitored throughout the process as previously described (Houghton et al., 2014).

The following experiments carried out:

i) 5.2 g Alginate bread (AB)
ii) 5.2 g Control bread (CB)
iii) 208 mg of MANUCOL DM alginate
iv) 5.2 g CB and 208 mg MANUCOL DM alginate

Model gut solution alone from 180 min was used as a control for all samples. 208 mg of alginate was used as this represents the total amount of alginate contained in 5.2 g of AB.

### 2.3 Viscosity measurements

Samples i–iv were added at the start of the model gut and the solutions were removed at the end of the model gut (180 min). Samples v was model gut solution from 180 min with 208 mg of Manucol^®^ DM and sample vi was the same solution taken from condition ii from 180 min with 208 mg of Manucol^®^ DM. In samples v and vi the alginate was added upon completion of the model gut procedure. All conditions were compared with model gut solution alone as a control. The heated alginates were measured at 2 mg/ml in distilled H_2_O, compared to distilled H_2_O alone.

Viscosity was measured using a Contraves low shear 30 viscometer at room temperature over the speed range of 10^−2^–10^2^ min^−1^, as previously described (Pearson & Roberts, 2001). Results were expressed as specific viscosity (without units as it was derived from viscosity of the sample divided by the viscosity of the solvent).

### 2.4 Heating of alginate

5 g of three sodium alginates (LFR 5/60, DM and SF200) with a molecular weight range of 40–380 kDa and a mannuronate:guluronate ratio of 0.44–1.38:1 were heated in pyrex tubes at 37, 100 and 200 °C for 30 min before being cooled to room temperature. DM alginate was also heated at 50 and 150 °C. These alginates were selected to observe the effects on alginates ranging in molecular weight and M and G content.

### 2.5 Freeze dried isolated alginate

Upon completion of the model gut incubation procedure (180 min) the solutions from 5.2 g AB, 5.2 g CB and 5.2 g CB with 208 mg MANUCOL DM alginate (i, ii and iv) were treated as follows. 4 ml and 8 ml samples of the 5.2 g AB and 5.2 g CB runs were prepared and a 4 ml sample of the 5.2 g CB with 208 mg DM alginate. All five samples were diluted by 50% with methanol, mixed and left at −20 °C for 30 min. The samples were then centrifuged at 4100 rpm for 20 min. The methanol was evaporated and the remnants were collected and the extraction repeated with a further 4 ml of methanol. The supernatants were combined and then freeze dried. The pellets for each sample were then weighed and the recovery of alginate calculated from the predicted alginate weight of each sample.

### 2.6 Gel filtration

A Sepharose 2B column 30 cm × 1.5 cm was eluted with sodium chloride (0.2 M) and sodium azide (0.003 M) at room temperature. 1 ml of each samples at a concentration of 1.43 mg/ml in elution buffer were loaded and 35 two ml fractions were collected. The samples subjected to gel filtration analysis were sodium alginates LFR 5/60, DM and SF200 post heating at 37, 100 and 200 °C. The freeze dried samples from the model gut, 5.2 g AB, 5.2 g CB and 5.2 g CB with 208 mg DM. 200 μl of each fraction were added to a 96-well plate in duplicate and the PAS assay was run as previously described (Houghton et al., 2014).

### 2.7 Lipase inhibition properties of heated samples and isolated alginate

This used the modified method of Vogel and Zieve (1961) and Wilcox et al. (2014). Orlistat 0.025 mg/ml in DH_2_O was used as an inhibition control. Heated DM alginate at 37, 100, 150 and 200 °C and freeze dried 5.2 g AB from the model gut were added to the substrate solution at 3 and 2 mg/ml and 3 and 2 mg/ml respectively.

To calculate the level of inhibition the following equation was used:

$$\text{Percent}\;\text{of}\;\text{lipase}\;\text{inhibition}=1-\frac{\text{Inhibition}\;\text{Control}-\text{Polymer}\;\text{Sample}}{\text{Inhibition}\;\text{Control}-\text{Lipase}\;\text{Control}}\times 100$$

### 2.8 Statistical analysis

Statistical calculations used SPSS Statistics 19 (IBM, Predictive Analysis Software, USA). Data presented as mean and standard error of mean (S.E.M). A Two-way Repeated ANOVA followed by a Post-Hoc Bonferroni were undertaken at a significant level (α) of 0.05 to compare the level of pancreatic lipase inhibition of DM alginate heated at 37, 100, 150 and 200 °C.

## 3 Results

### 3.1 Viscosity of model gut samples

**Fig. 1:**
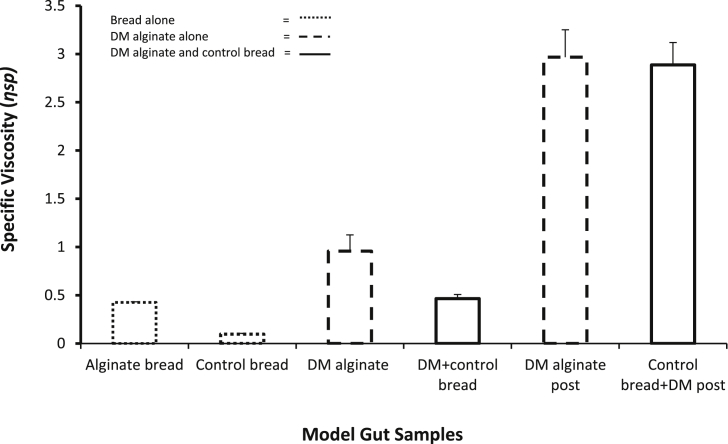
Mean (+S.E.M.) viscosity of samples taken from the end of the model gut t = 180. Alginate Bread (5.2 g), Control Bread (5.2 g), DM alginate (208 mg) and 5.2 g CB with DM alginate (208 mg) were added at beginning of model gut. DM alginate (208 mg) was added to model gut solution and 5.2 g CB solution from the end of the model gut. GMS is gut model solution (n = 6).

The viscosities of the samples from the model gut are shown in Fig. 1. The specific viscosity of the control bread solution after passing through the model gut (t = 180 min) was low 0.11 ± 0.01 as expected the viscosity of the alginate bread at t = 180 min was higher by a factor of 4 (0.42 ± 0.01). DM alginate added at time zero, at the concentration as would be released from the alginate bread at 180 min had a higher viscosity than the alginate bread i.e. 0.91 ± 0.41. Control bread with DM alginate added at time zero had a viscosity of 0.46 ± 0.11 similar to that for alginate bread. These data suggest that when bread is present there is a reduction in the ability of alginate to form a viscous solution. The viscosity of DM alginate added to model gut solution at the end of the procedure (t = 180) was 2.96 ± 0.71 and the viscosity of DM alginate added to the control bread that had passed through the model gut was 2.88 ± 0.57. These results demonstrate that alginate passing through the model, either in a bread vehicle or free had a lower specific viscosity than if added to solution from the model gut once it has finished. The combination of the bread and model gut process attenuates the ability of the alginate to form a viscous solution.

### 3.2 Viscosity of heat treated alginate

The data presented in Table 2 is for alginates LFR 5/60, DM and SF200 at 37 °C and post heating at 200 °C. The specific viscosity relates to the molecular weight with SF200 having a specific viscosity of 14, DM alginate 12 and LFR 5/60 2.2. Post heating at 200 °C there was an almost complete loss of viscosity. These data indicate extensive fragmentation of the alginate chains following heating at 200 °C.

**Fig. 2:**
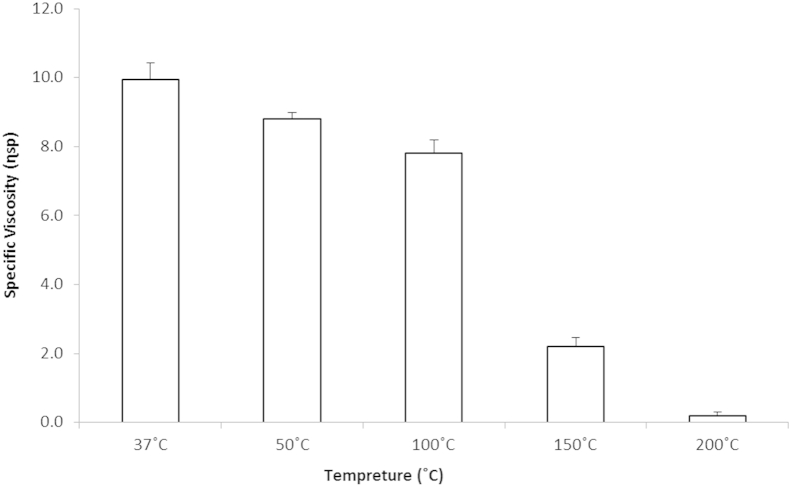
Mean (±S.E.M.) *ηsp* of DM alginate after being heated at 37, 50, 100, 150 and 200 °C for 30 min. Each sample was then allowed to return to room temperature and then re-suspended in DH_2_O at 1.43 mg/ml (n = 6).

The *ηsp* for heated DM alginate is illustrated in Fig. 2. The *ηsp* remained relatively stable between 37, 50 and 100 °C, with only a reduction of 16 and 21% for 50 and 100 °C. Beyond 100 °C there is a large drop in *ηsp*. The *ηsp* for 150 and 200 °C was 2.2 and 0.2 respectively, equating to a reduction of 78 and 99%. The data in Fig. 2 indicate exposure to high temperatures alter the ability of alginate to form viscous solutions.

### 3.3 Freeze dried samples isolated from the end of the model gut

**Fig. 3:**
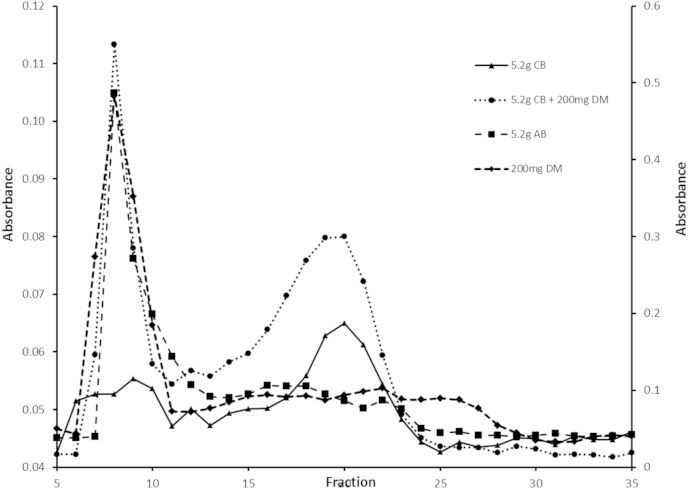
Mean (±S.E.M.) ■ = elution profile from freeze dried 5.2 g AB, ▴ = 5.2 g CB, ● = 5.2 g CB and 208 mg and ♦ = 208 mg DM alginate alone from the end of model gut and isolated process. Freeze dried samples were then re-suspended in DH_2_O at 1.43 mg/ml based on the freeze dried weight and processed through the gel filtration using Sepharose 2B. PAS assay was used to quantify alginate in fractions collected from gel filtration. Vertical axis 1 is for AB and vertical axis 2 is for CB alone and CB with DM alginate (n = 6). V_o_ and V_t_ from calibration are identified with fractions 8 and 32 using dextran blue and methyl orange respectively (n = 6).

The alginate fraction isolated from the model gut solution at the end of processing alginate bread in the model gut had a major PAS positive peak eluting at the excluded volume, where DM alginate elutes there is a tail of material stretching into the included volume (Fig. 3). The control bread with an alginate spike at time zero, had a major peak at the excluded volume with a second substantial peak in the included volume. Control bread alone had a small excluded peak and a peak in the included volume in a similar position (i.e. fraction 20) to the included peak of the control bread spiked with alginate. These results indicate that even after cooking alginate bread retains high molecular weight alginate.

### 3.4 Lipase inhibition by isolated and heated alginate

**Fig. 4:**
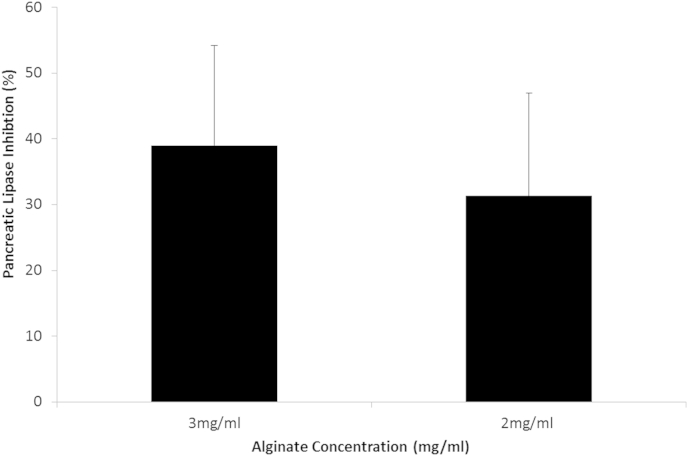
Mean (±S.E.M.) pancreatic lipase inhibition with isolated alginate from end of the model gut and following freeze drying. Freeze dried AB were re-suspended in lipase buffer at 3 and 2 mg/ml and the olive oil turbidity assay was run (n = 9).

The alginate extract from the end of the model gut was re-suspended in substrate solution to ascertain whether the alginate is able to retain its inhibition properties post cooking into the bread, digestion and isolation. The freeze dried extracts from 5.2 g AB inhibited pancreatic lipase by 39 (±0.42) and 36 (±0.43) at 3 and 2 mg/ml respectively (Fig. 4). These data indicate that although the alginate has been cooked into the bread, digested in the model gut and isolated it retains inhibition properties.

**Fig. 5:**
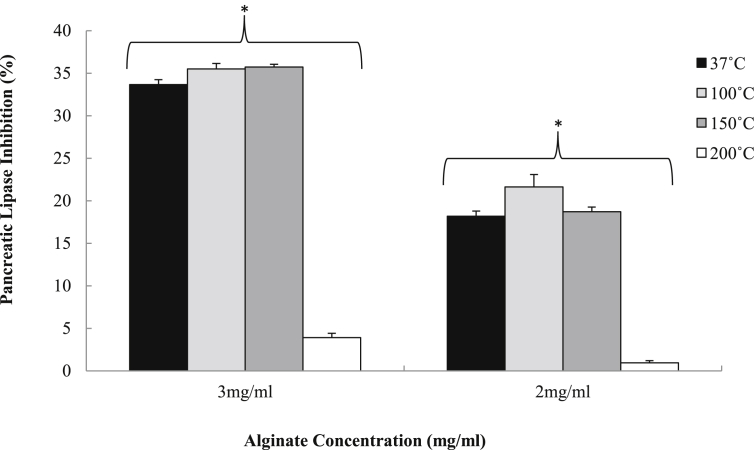
Mean (±S.E.M.) pancreatic lipase inhibition using DM alginate after it has been heated at 37, 50, 150 and 200 °C for 30 min. After being left at room temperature the DM alginate was then re-suspended in lipase substrate solution at 3 and 2 mg/ml and the turbidity assay was performed (n = 9). *Denotes a significant difference (<0.05), between temperatures 37, 100, 150 and 200 °C at 3 and 2 mg/ml.

Heated DM alginate at 3 and 2 mg/ml inhibits pancreatic lipase. These data indicate that there is a significant effect for concentration and temperature (p < 0.05). The level of inhibition for 37 °C was 33% (±0.58) and 18% (±0.57), 100 °C was 35 (±0.64) and 21% (±1.46) and 150 °C was 35% (±0.32) and 18% (±0.55) for 3 and 2 mg/ml respectively. The ability to inhibit lipase was all but abolished by heating to 200 °C with 4% (±0.48) and 1% (±0.26) for 3 and 2 mg/ml respectively when heated at 200 °C. There was no significant difference between temperatures 37, 100 and 150 °C for matched concentrations at 3 and 2 mg/ml (p > 0.05). The DM alginate heated at 200 °C was significantly different from temperatures 37, 100 and 150 °C at concentration 3 and 2 mg/ml (p < 0.05). The data in Fig. 5 indicate that DM alginate is able to inhibit pancreatic lipase despite being heated at 150 °C.

## 4 Discussion

The ability of alginates to form both acidic and ionic gels may be responsible for poor mixing within the stomach and small intestine, and ultimately this will interfere with the ability of digestive enzymes to interact with substrates and attenuate nutrient digestion (Georg Jensen, Kristensen, Belza, Knudsen, & Astrup, 2012; Smidsrod, 1974). Paxman, Richardson, Dettmar, and Corfe (2008) and Hoad et al. (2004) stated that if the alginate forms a viscous solution/gel in the stomach this may cause distension within the stomach and increase satiety, and therefore reduce the number of calories an individual may consume. This was evident in previous research that have used alginate in a beverage and shown that alginate can modulate fat metabolism (Sandberg et al., 1994), however we have used a cooked product and needed to ascertain if the cooking process effected alginate properties.

Previously presented data has indicated that when AB is digested between 10 and 15% of alginate is released in the stomach with the remaining 85–90% released in the small intestine (Houghton et al., 2014). Therefore it seems reasonable to assume that if all the alginate is in fact being released, then the viscosity of the solution at the end of the model gut should increase. However there is not a substantial increase in viscosity from the digested AB. A specific viscosity of 0.42 ± 0.01 is much lower than the viscosity that would be expected for a similar concentration of alginate i.e. 8–10 in distilled water pH 7.0 for 1.43 mg/ml DM alginate. For this reason alginate was added at the start of the model gut alone and at the end to see if the model gut had any effect on viscosity. Alginate added to the start of the model gut and processed through the model gut had a viscosity about twice that of the alginate released from the bread. This was only about 1/3 of the viscosity generated by alginate added at the end of the model gut T = 180. These data suggest that the alginate may be degraded or be binding to other components within the model gut (Adiotomre, Eastwood, Edwards, & Brydon, 1990; Wang, Onnagawa, Yoshie, & Suzuki, 2001). An explanation for the drop in viscosity is pH dependent degradation. In the gastric phase of digestion the pH falls to around 2 and proton catalysed hydrolysis can occur (Draget, 2000, chap. 29). In addition commercial alginate can contain small amounts of phenolic compounds which can catalyse oxidative-reductive depolymerisation (Draget, 2000, chap. 29). In fact subjection of alginate in DH_2_O to the changes in pH for the same time course as the model gut results in a fall in viscosity from 9.9 to 2.8. Any further loss of viscosity can be attributed to hyperosmolarity in the model gut reducing the hydrodynamic size of the alginate and fragmentation caused by cooking. A further possibility is an interaction between alginate and bile acids reducing viscosity. Wang et al. (2001) reported that both the soluble and insoluble forms of dietary fibre from seaweeds were able to bind bile acids, although soluble dietary fibres were significantly better. This explanation is unlikely because the final viscosity of alginate processed through the model gut was the same if pig bile was included or omitted (data not presented). It cannot be ruled out that some interaction is taking place between the bread components and alginate. This could explain the 50% difference in viscosity between DM alginate processed through the model gut and processed through with control bread.

During the cooking process the bread may be subject to temperatures up to 200 °C for 30 min. Although the exact temperature of the entire loaf of bread here cannot be determined, Hasatani et al. (1991) observed the effects of various bread recipes and measured the temperature of different parts of the bread. They indicated that the centre of the bread may only reach 70–80 °C, however the crust may be exposed to between 180 and 200 °C. It was apparent that there is a substantial loss in viscosity ranging between 92 and 98% when alginate is exposed to 200 °C. In contrast temperatures up to 100 °C had little effect. Suggesting that alginate towards the centre of the bread would be unaffected and that in the crust fragmented resulting in a mixture of different size alginates being released from the bread on digestion. These data are in agreement with the work of McDowell (1977) who demonstrated that as alginates in solution are exposed to temperatures above 100 °C a gradual depolymerisation occurs until complete breakdown of the polymer. This breaking down of the polymer may account for the reduction or complete lack of viscosity as reported here. Larger alginates have a larger Young's modulus (Smidsrod, 1974), meaning they can form viscous gels. Consequently if the size of the alginate is being reduced during the cooking process then this will impact upon the alginate's ability for form a gel. This size change was confirmed by gel filtration (data not shown). An additional point of interest may be the molecular composition of the alginate used. There is currently no research comparing the heat stability of a range of alginates varying in gluronate:gluronate (GG) residues, mannuronate:mannuronate (MM) residues and a mixture of gluronate:mannuronate (GM) residue blocks. It is well known that the properties of GG blocks are different from GM and MM blocks in gel formation. This therefore raises the question as to whether alginates with a larger GG content are more or less heat stable than a combination of MG and MM blocks. If the alginate is affected by the heating during the cooking process then the isolated alginate from the digested alginate bread would have a reduced hydrodynamic size on gel filtration. However the majority of the released alginate was excluded on the column indicating it was still relatively large. A longer column would have allowed a better analysis of the excluded material to see if it contained several different sized species as a result of limited degradation. It appears that the bread matrix is protecting the alginate from the high temperatures or the temperature is not that high inside the bread.

If the alginate cooked into the bread is able to withstand cooking and digestion then it would appear that the bread may indeed be a suitable vehicle to add alginate to an individual's diet. Despite being robust to withstand these processes the question remains as to whether the alginate retains its inhibition properties once cooked into the bread, despite the reported reduction of viscosity. Wilcox et al. (2014) and Richardson, Dettmar, Wilcox, Brownlee, and Pearson (2011) have reported that certain alginates are able to reduce the activity of pancreatic lipase by up to 75%, which is dependent on the structure of the alginate. The method used here relies on a turbidity assay using olive oil as a substrate. This assay has been extensively validated and gives comparable results to a lipase activity assay using 1,2, Di-o-lauryl-rac-glycero-3-(glutaric acid 6-methyl resorufin ester) (DGGR) as a synthetic substrate (Wilcox et al., 2014). In addition the activity of the lipase is quoted by the manufacture against olive oil. The data from the present study reveals that DM alginate retains its inhibition properties after being cooked, digested, isolated or heated at 150 °C. These data not only support the work of Asp (1987) but also demonstrates the robustness of alginate after being processed in the various methods described here. There was no significant difference at temperatures 37, 100 and 150 °C at matched concentrations for lipase inhibition despite the reduction in viscosity at these temperatures particularly at 150 °C. Upon exposing the alginate to 200 °C the alginate appeared to lose 88% of its inhibition properties. The data presented here in accordance with previous work indicates that alginates undergo depolymerisation when exposed to temperatures above 100 °C, which has a profound detrimental impact upon gel formation. Interestingly, when exposed to 150 °C the Manucol^®^ DM alginate lost 78% of its *ηsp* when compared with 37 °C, however there was no such reduction when observing the effect of heat on alginates ability to inhibit pancreatic lipase activity. Conversely there was a reduction of 98 and 88% for *ηsp* and pancreatic lipase inhibition respectively, when exposed to 200 °C. These data suggest that there is substantial alteration in polymer structure at temperatures beyond 150 °C which is essential for gel formation (Braccini & Perez, 2001; Draget, Stokke, Yuguchi, Urakawa, & Kajiwara, 2003), and inhibition properties (Richardson et al., 2011; Strugala et al., 2005; Wilcox et al., 2014). There is little data on other fibres enclosed in a food matrix and their ability to inhibit pancreatic lipase. However there is evidence that other fibres have lipase inhibition properties, such as pectins (Isaksson, Lundquist, & Ihse, 1982) and chitosan (Han, Kimura, & Okuda, 1999).

There is evidence to suggest that viscosity may play a crucial role in the reduction of enzyme activity (Seal & Mathers, 2001; Shah, Mahoney, & Pellett, 1986), however the data here suggests that this is not the case for lipase inhibition. There are other mechanisms which may also play a role including substrate binding (Strugala et al., 2005; Wilcox et al., 2014). The data presented shows alginates with a low *ηsp*, are still able to inhibit pancreatic lipase activity by up to 38%. The mechanism/s of how alginate inhibits pancreatic lipase is at present not elucidated but it could be like in pectins which are believed to protonate the active site serine and histidine residues (Kumar & Chauhan, 2010). Although this data is positive the question remains as to whether the AB will be able to inhibit pancreatic lipase *in-vivo*. The turbidity assay used here was adapted from Vogel and Zieve (1961) and works on the basis that as the fat is digested the substrate solution becomes more transparent. Although this is an effective method for determining pancreatic lipase activity, this does not however take into account other factors within the in vivo gut such as bile, undigested bread and the plethora of other enzymes involved in digestion. Further work is required to ascertain whether the lack of viscosity reported here has any impact upon the ability of AB to inhibit, fat digestion *in-vivo*. In conclusion the present study demonstrates that alginates are heat stable up to temperatures of 100 °C. However beyond 100 °C there is a gradual decrease in viscosity as the temperature increases. By contrast the ability of the alginate to inhibit pancreatic lipase appears to be unaffected up to temperatures of 150 °C, despite the reduction in viscosity and by implication size. Furthermore, despite being used as an additive to a bread vehicle, cooked, digested in a model gut and isolated the alginate retains its inhibition properties and does not appear to be affected by these processes.

## Figures and Tables

**Table 1:** Greggs Plc bread ingredients with or without Manucol^®^ DM alginate at 4% per 100 g.

Regular bread	BF0003-5	
**Nutrients per**		**100 g**
Energy (kcal)		247
Energy (kJ)		1046
Protein (g)		10.2
Carbohydrates (g)		46.2
	Sugars	1.1
	Starch	45
Fat (g)		1.7
	Saturates	0.5
	Monosaturates	0.3
	Polyunsaturates	0.6
	Trans	0
Dietary fibre (AOAC) (g)		3
Sodium (g)		0.4 (374 mg)
Water (g)		36.8

**Table 2:** Mean (±S.E.M.) specific viscosity of alginates at 2 mg/ml in DH_2_O at 37 °C and post heating at 200 °C for 30 min (n = 6).

Alginate	Molecular weight	Pre heating 37 °C	Post heating at 200 °C	% Δ in *ηsp*
DM	250,000–320,000	12.0 (0.25)	0.1 (0.07)	99.6
LFR 5/60	40,000	2.2 (0.19)	0.2 (0.01)	90.1
SF200	380,000	14.0 (0.08)	0.2 (0.01)	98.9
